# Scientific Inquiries with Reference to the Yellow Fever Epidemic of 1888, in Jacksonville, Fla.

**Published:** 1889-09

**Authors:** Joseph Jones

**Affiliations:** New Orleans, Louisiana


VOL. VI. SEPTEMBER, 1889. NO. 7.

### Original Communications.

### SCIENTIFIC INQUIRIES WITH REFERENCE TO THE YELLOW FEVER EPIDEMIC OF 1888, IN JACKSONVILLE, FLORIDA.*

ADDRESSED BY JOSEPH JONES, M. D., OF NEW ORLEANS, LOUISIANA, TO SEVERAL PHYSICIANS ENGAGED IN ITS TREATMENT, WITH REPLIES THERETO.

156 WASHINGTON STREET, FOURTH DISTRICT,
NEW ORLEANS, LOUISIANA, October 8, 1888.

DEAR DOCTOR :—Please excuse the liberty I take in asking you to give me, at your earliest convenience, a statement of the following facts :

1. Origin of the epidemic of yellow fever in 1887-'88 ?

2. Cases illustrating the changes of temperature, pulse, respiration, secretions and excretions in the epidemic?

3. *Post mortem* examinations ?

*Read before the Eleventh Annual Meeting of the Louisiana State Medical Society, New Orleans, April 10, 1889.

4. Relative proportion of cases to whole population? To whites? To blacks? To colored?

5. Relative proportion of deaths to whole population? To whites? To blacks?

6. Relative proportion of deaths to cases? Relative mortality of natives and foreigners? Relative mortality of whites and blacks? Races.

7. Modes of treatment? What was the most scientific mode of treatment? Was the microbe of yellow fever discovered and isolated?

8. Was inoculation practiced and with what result?

9. Were prophylactics, as sulphite and hypo-sulphite of soda, quinine, etc., used to any extent, and with what results? Is it possible to prevent the occurrence of yellow fever by the use of external remedies, or is it possible to modify the progress of the disease by any known agent?

10. What disinfectants were used? Had disinfectants and sanitary measures any effect in controlling the spread of, or modification of, the virulence of the disease?

Respectfully,

Your obedient servant,

JOSEPH JONES, M. D.

UNITED STATES MARINE HOSPITAL SERVICE,
DISTRICT OF THE GULF,
CAMP PERRY HOSPITAL, October 22, 1888.

*Professor Joseph Jones, New Orleans:*

DEAR PROFESSOR:—Your honored letter of the 8th reached me but yesterday at Camp Perry, where I have charge of the hospital for the Marine Hospital Service. I am sorry to be unable for the present to give you the data requested, but shall be happy, if I return through Jacksonville, to try to obtain them from the President of the Board of Health. My experience this year has been very limited, having taken about thirty observations of pulse and temperature; but this experiment of a detention camp inland, which I believe a new departure in the history of epidemics, is very interesting. A refuge camp should be added for those who do not wish to go far away. About eight hundred refugees have been detained ten days at the Camp, on their way North. Only thirty have developed cases of yellow fever, several being sick already before leaving Jacksonville. None of the refugees who left the Camp, after this detention of ten days, have been known to develop a case elsewhere. Only one case terminated fatally here, at the start of the Camp before I took charge of the Hospital, and the cause of death could be ascribed to delirium tremens, rather than to yellow fever. The treatment under hospital tents and in rough open houses seems favorable. I give 10 to 20 drops of tincture muriate of iron four times a day, large quantities of milk with bicarbonate of soda, and calcined magnesia (Husband's) at the start and sustaining measures. I believe hyper-purgation and overheating blankets injurious. Why should not the muriate of iron have the same happy effects in yellow fever as it has in diphtheria, erysipelas and typhoid fever? A chloride not dangerous. Is it not the best remedy in black vomit? Dear doctor, please excuse this long flow of words from one of Your respectful pupils,

C. FAGET, M. D.

CORRESPONDENCE WITH REFERENCE TO THE CASE OF PROF. R. A. PROCTOR, THE EMINENT ASTRONOMER—TESTIMONY OF DR. GEORGE TROUP MAXWELL—REPLY OF JOSEPH JONES, OF NEW ORLEANS.

63 LAURA STREET, JACKSONVILLE, FLA., February 16, 1889.

MY DEAR SIR:—I take the liberty to enclose to you the report of the doctors who made a *post mortem* examination of my friend, the late Mr. Proctor, and I ask that you will give me your opinion upon the same.

I will be pleased to have the benefit of your great experience upon the report as a whole, but especially with regard to the statement that "the alterations produced in the body by yellow fever are usually of such a character as to be nearly or completely obliterated by advanced decomposition."

I have been requested by the family and friends of Mr. Proctor to make investigation of the facts of his case for publication.

The plantation and grove, which was improved and beautified by my wife, now belongs to her son, Mr. Geo. W. Wilson. Two years ago, Mr. Wilson gave Mr. Proctor ten acres of land within three hundred yards of the oak lawn, upon which he erected a handsome cottage, in which he lived for about a year. The place is eighteen miles from Ocala, and is in the country, as much isolated as any country home in Florida. He had not left his place for months except to walk half a mile to a picnic in a ———————, when he stayed about thirty minutes, having been sick with intermittent fever the day before. He was again attacked by a paroxysm of the same fever the next day, Friday. Started North Saturday, during a second intermission; had another paroxysm Sunday *en route;* arrived in New York Monday, during the relief of another intermission, and was taken early Tuesday morning with the last and a fatal paroxysm, when his illness assumed the nature of a malignant, contagious attack of malarial hæmaturia. This produced the consternation among the hotel employees and the New York doctors. The panic-fright extended to editors and reporters, and this good and great man was hustled out of comfortable quarters in a rain storm in the dead of night, rolled over most uneven pavements to the Willard Parker Hospital, and to his death.

Besides the history of his illness as concisely given, showing that he died not from yellow fever, it is susceptible of incontrovertible proof that he was not exposed to the infection of yellow fever either at home or on the way to New York.

In this case Florida has been slandered, science has been dishonored, and the life of a brilliant scientist and man of letters has been jeopardized, if not sacrificed, by what the *Courier-Journal* called the "Cruelty of Cowardice."

Your words will be authoritative and conclusive, therefore I beg that you will express them.

Very truly your friend,

(Copied.) GEO. TROUP MAXWELL.

The following is the extract from the report of "the Pathologists to the health department of New York City," enclosed by Dr. Maxwell, of Jacksonville, Florida.

"In accordance with your request an autopsy on the body of Prof. R. A. Proctor was made by us at 2:30 P. M. to-day, at North Brother Island, and we submit the following preliminary report:

"Decomposition was so far advanced that it was impossible to arrive at a definite conclusion as to the cause of death. The organs presented no evidence whatever of pernicious remittent fever or other form of malarial disease, such as, even in the condition of decomposition presented by the body, would ordinarily be apparent.

"The only positive change due to disease which could be made out, was in the kidneys, which had the appearance of old, though not advanced disease.

"The alterations produced in the body by yellow fever are usually of such a character as to be nearly or completely obliterated by advanced decomposition. We are, therefore, only able to say in this connection that there was no other evident cause of death, and nothing that would be incompatible with death from that disease.

"The final conclusion as to the cause of death must therefore, in our opinion, be largely based upon the clinical history."

T. MITCHELL PRUDEN, M. D.
HERMAN M. BIGGS, M. D.
Pathologists to Health Department of New York City.

*New York, September 16.*

156 WASHINGTON STREET, CORNER OF CAMP ST.
(4th District.)
NEW ORLEANS, LA., February 20, 1889.

DR. GEO. TROUP MAXWELL,
63 Laura Street, Jacksonville, Fla.

MY DEAR DOCTOR:—I take pleasure in replying to the request contained in your kind and valued favor of February 16th, 1889, and respectfully submit the following:

1st. The report of the *Pathologists to the Health Department of New York City is incomplete, vague and unsatisfactory.*

This proposition will be sustained by the following considerations:

(a) The size of the organs is not given.

(b) The color of the organs is not even alluded to.

(c) The report contains no evidence to show that any microscopical or chemical analysis of the organs of Prof. Proctor were made by the "*Pathologists to the Health Department of New York City.*"

2d. The statement of the "Pathologists to the Health Department of New York City," that "the alterations produced in the body by yellow fever, are usually of such a character as to be nearly or completely obliterated by advanced decomposition," is not only indefinite but contains evidence of neglect of duty on the part of the health authorities of New York.

Our second proposition will be sustained by the following considerations:

(d) The yellow fever poison induces acute fatty degeneration and change of the structures of the heart and liver. The accumulation of oil globules in the structures of the heart and liver can be determined, even during advancing and advanced decomposition, by microscopical examinations and chemical analyses. In uncomplicated malarial fever there is no increment of oil, either in the cardiac or hepatic structures. In every doubtful, or so-called *suspicious* case of fever, the organs, and especially the *heart* and *liver* and *kidneys*, should be subjected to rigid microscopical and chemical analyses and examinations. No such examinations appear to have been made in the case of Professor Proctor.

(e) Why was the body of Professor Proctor allowed, by the health authorities of New York, to proceed to such "*advanced decomposition?*"

In genuine yellow fever, the liver, infiltrated with oil globules, decomposes with comparative slowness and the characteristic fatty degeneration may be determined by both chemical and microscopical investigation for long periods after death. Why did the health authorities of New York allow a sufficient period for the occurrence of advanced decomposition in the body of

Professor Proctor before performing the necessary autopsy and chemical and miscropical tests to determine the true nature of this disease ? If measures are to be instituted for the arrest of yellow fever, they should be inaugurated at once.

The first step in any intelligent system of defence against the spread of such contagious diseases, as Asiatic cholera and yellow fever, is the formation of an accurate diagnosis based upon thorough clinical records and exhaustive *post mortem* examinations, performed immediately after death, and before decomposition has progressed to such an extent as to render the indications of diseased processes obscure.

3d. The pathologists of the New York health department, state that "the only positive change due to disease which could be made out was in the kidneys, which showed the appearance of old, though not advanced disease." We find it to be impossible to form any definite idea as to the meaning or application of this indefinite statement.

In what specific disease are the terms " old but not advanced," applicable ? It is true that the kidneys are frequently involved in yellow fever, and that albumen frequently occurs with granular casts in the urine. It is true that the kidneys, after death from yellow fever, present pathological alterations recognizable by the naked eye and by the microscope.

It is equally true that the kidneys present marked pathological alterations in malarial hæmaturia, scarlatina and the various forms of Bright's disease.

4th. From the preceding analysis, we conclude, that there are no facts stated by the pathologists of the health department of New York City to show that the death of Professor Proctor was caused by yellow fever.

With your permission, I will present this correspondence at the meeting of the Louisana State Medical Society, on the 9th of April, 1889.

I also respectfully and earnestly beg that you will, at the earliest practicable moment, prepare a paper giving your views as to the origin and nature of the recent epidemic of yellow fever in various localities of Florida; also your plan of treatment with statistical results.

I desire to present your paper to our Medical Society at its meeting in April.

With kind regards, truly your friend,

JOSEPH JONES, M. D.

The following letter and accompanying report were received from Dr. George Troup Maxwell on the 26th of March, 1889.

*Professor Joseph Jones, M. D., New Orleans, La.:*

DEAR DOCTOR:—I have just succeeded in obtaining the full report of the autopsy made upon the body of Mr. Proctor. I send it for you to examine.

Have you any additional remarks to make upon this interesting case? If so please let me have them and return this report at the same time. Very respectfully, your friend,

GEORGE TROUP MAXWELL.

REPORT OF THE AUTOPSY ON THE BODY OF PROFESSOR RICHARD PROCTOR, AT NORTH BROTHER ISLAND.

Present—Drs. Bryant, Jacobi, Edom, Conant, Priest and others.

The body was fairly well nourished. The conjunctivæ of a slight yellow color. Decomposition, staining of the tissues along the line of the superficial veins of the chest.

*Rigor mortis* absent.

*Intestines.* Moderately distended with gas, peritoneal cavity apparently normal. Diaphragm at 4th rib, right side; at 5th in left side.

*Liver.* Lies considerably above the edge of ribs on right side. Rib cartilages moderately calcified. A few old pleuritic adhesions on the right side above. About three ounces dark red fluid in each pleural cavity.

*Heart.* Pericardium normal. The cavities of the heart on both sides are empty. The heart muscle was flabby from decomposition. The edge of the mitral valve was slightly thickened. The endocardium was deeply stained from decomposition of the blood. There was a slight amount of fatty degeneration of the aorta, near the aortic valves. Slight endarteritis of the main branches of the coronary arteries.

*Lungs.* Left, pleura normal; hypostatic congestion of the lower part of the upper lobes, and the entire lower lobe. Right, emphysema; hypostatic congestion of the lower lobe.

*Spleen.* Weight twelve ounces. No evidence of old interstitial inflammation, dark red in color, almost diffluent from decomposition.

*Liver.* Weight two pounds, eleven ounces. Color, dark from *post mortem* staining. Cut surface uniform in appearance. Tissue soft and flabby from decomposition. The gall-ducts were patulous.

*Kidneys.* Right, almost normal in size. A few small irregular depressions scattered over the surface. The whole organ soft and diffluent. Left presented the same appearance as the right.

The *Stomach* and *Intestines*, so far as could be judged in the advanced stage of decomposition, were essentially normal, save for a few old adhesions and small diverticulæ of the colon.

The *Bladder*, containing about six ounces of reddish yellow turbid fluid, appeared normal. The prostate was slightly enlarged. The conclusions which could be drawn from the results of the autopsy in the advanced stage of decomposition have been already given in the preliminary report.

Respectfully submitted,

HERMAN H. BIGGS, M. D.,

J. MITCHELL PRUDEN, M. D.,

Pathologists to the Board of Health of New York City.

156 WASHINGTON STREET, NEW ORLEANS, March 26, 1889.

*George Troup Maxwell, Jacksonville, Florida:*

DEAR DOCTOR:—In accordance with your request, I have read the report of Drs. T. Mitchell Pruden and Herman H. Biggs, pathologists to the board of health of New York City, of the autopsy of the body of Professor Richard Proctor; and beg leave to submit the following in addition to my former observations.

First. The report of the autopsy is without date. The time which elapsed between the death of Professor Proctor and the time of performing the autopsy is not given. The extent of decomposition, is in the heat of the summer, proportion mode in which the body was preserved by embalming or by refrigeration and to the time after death. In what manner was the body of Professor Proctor preserved after death? The report is silent on this important subject.

Second. The report is silent as to the color of the body of Professor Proctor. The only remark bearing on this question being *the conjunctivæ of a slight yellow color*. As a rule the surface of the body as well as the conjunctivæ of the eye, present a deep, golden color after death from yellow fever.

Third. The weight of the liver is stated to have been two pounds, eleven ounces; this is below the average weight in health. Weight of healthy liver, mean of eighty-two observations in males, three to four pounds. "Surface *dark* from *post mortem* staining; cut surface uniform in appearance; tissue soft and flabby from decomposition." It does not appear that the liver was subjected to chemical or microscopical examinations, and there is nothing in the superficial observations to indicate anything farther than that the organ was in a decomposing state.

Fourth. We have no information as to the condition of the gall bladder, whether empty or distended, and no observation as to the quantity, color and chemical composition of the bile. The report simply says that the gall ducts were patulous. In most cases of malarial fever the gall bladder is distended with more than one thousand grains of thick, greenish, black bile; in yellow fever, on the contrary, the gall bladder is contracted, flaccid, small, and contains little or no bile. The amount of bile generally does not exceed 100 grains.

Fifth. The weight of the spleen, twelve ounces, is above the normal weight of the organ in health, which varies from five to seven ounces. In yellow fever the spleen, as a general rule, is but slightly enlarged, and in many cases is normal in size and appearance, being neither enlarged nor softened. On the other hand in malarial fever the spleen is enlarged, softened and loaded with blood corpuscles and pigment granules, and of a dark slate color on the exterior. In many cases the spleen is so soft that it ruptures when the attempt is made to remove it from the abdominal cavity. The report describes the spleen of Professor Proctor as *dark red* in color and almost diffluent from decomposition. This is the description of the spleen in malarial fever.

Sixth. We gather from the report that the heart and kidneys indicated advanced putrefaction, but gave no evidence of any characteristic pathological lesion.

Seventh. The stomach presents marked pathological alterations in yellow fever and contains black vomit in greater or less quantities after death from this disease, and yet the statement is made that in the case of Professor Proctor, "*the stomach and intestines*, so far as could be judged in the advanced stage of decomposition, were essentially normal."

Eighth. No chemical or microscopical analysis is given of the "*six ounces* of *reddish*, *yellow*, *turbid* fluid" contained in the bladder. In yellow fever the urine is in fatal cases scant, and contains albumen and casts. In malarial hæmaturia the urine contains blood corpuscles, hæmoglobin and casts.

In conclusion, we fail to find in this report any facts indicating that Professor Proctor's death was caused by yellow fever.

With kind regards, Truly your friend,

JOSEPH JONES, M. D.

